# Rationale for the Use of Cord Blood in Hypoxic-Ischaemic Encephalopathy

**DOI:** 10.1155/2022/9125460

**Published:** 2022-05-11

**Authors:** Izabela Zdolińska-Malinowska, Dariusz Boruczkowski, Dominika Hołowaty, Paweł Krajewski, Emilian Snarski

**Affiliations:** ^1^Polski Bank Komórek Macierzystych S.A. (FamiCord Group), Jana Pawła II 29, 00-86 Warsaw, Poland; ^2^Department of Obstetrics and Gynecology, Medical University of Warsaw, Starynkiewicza Square 1/3, 02-015 Warsaw, Poland; ^3^Department of Experimental and Clinical Physiology, Laboratory of Centre for Preclinical Research, Medical University of Warsaw, Warsaw, Poland

## Abstract

Hypoxic-ischaemic encephalopathy (HIE) is a severe complication of asphyxia at birth. Therapeutic hypothermia, the standard method for HIE prevention, is effective in only 50% of the cases. As the understanding of the immunological basis of these changes increases, experiments have begun with the use of cord blood (CB) because of its neuroprotective properties. Mechanisms for the neuroprotective effects of CB stem cells include antiapoptotic and anti-inflammatory actions, stimulation of angiogenesis, production of trophic factors, and mitochondrial donation. In several animal models of HIE, CB decreased oxidative stress, cell death markers, CD4+ T cell infiltration, and microglial activation; restored normal brain metabolic activity; promoted neurogenesis; improved myelination; and increased the proportion of mature oligodendrocytes, neuron numbers in the motor cortex and somatosensory cortex, and brain weight. These observations translate into motor strength, limb function, gait, and cognitive function and behaviour. In humans, the efficacy and safety of CB administration were reported in a few early clinical studies which confirmed the feasibility and safety of this intervention for up to 10 years. The results of these studies showed an improvement in the developmental outcomes over hypothermia. Two phase-2 clinical studies are ongoing under the United States regulations, namely one controlled study and one blinded study.

## 1. Introduction

Neonatal hypoxic-ischaemic encephalopathy (HIE), which occurs in 2–3 per 1000 newborn children [1], is one of the leading causes of death in newborns (6–9%) and infants (21–23%) worldwide, with an incidence exceeding 1 million deaths per year [2]. The consequences of HIE vary depending on the severity of ischaemia, sex, and therapeutic intervention. HIE results from the disruption of cerebral blood flow (ischaemia) and oxygen delivery (hypoxia) to the brain during the perinatal period. The direct consequence of ischaemic insult is the inhibition of oxidative phosphorylation, deficiency of adenosine triphosphate, decreased activity of adenosine triphosphate- (ATP-) dependent ion pumps, and failure of cell membrane ion pumping. Consequently, this results in the flow of sodium and calcium ions accompanied by water into the cell, which leads to progressive cell swelling and necrosis [3]. Membrane depolarisation opens voltage-sensitive calcium channels and results in calcium influx, leading to the production and release of glutamate [3]. This excitatory amino acid activates receptors which further promote the influx of calcium ions into cells. Reperfusion contributes to further injuries caused by intensive production of free radicals, resulting in secondary ATP depletion and subsequent apoptotic brain damage [4]. Additionally, oxygen deprivation, energy depletion, and reoxygenation lead to the generation of reactive oxygen species, which cause oxidative damage to proteins, nucleic acids, and membrane lipids, thereby disturbing their function [5]. Oxidative stress leads to mitochondrial impairment and rapid activation of microglia, followed by infiltration of mobilised circulating leukocytes, monocytes, and neutrophils [6–9]. After entering the central nervous system, microglia release cytokines and chemokines, increasing inflammation. Late consequences include altered synaptogenesis, delayed oligodendrocyte maturation, and epigenetic dysfunction in the surviving cells [10]. The entire process has recently been described in detail by other authors [11–15].

Some researchers have described sexual dimorphism in microglial numbers and expression of activation markers in the neonatal brain under both normal and hypoxic conditions; males are more vulnerable to brain damage caused by HIE. In the first study, female mouse neonates, which were a model of HIE, had significantly smaller infarct sizes, fewer seizures, and less brain tissue loss and behavioural deficits, whereas males had increased microglial activation and an upregulated inflammatory response [16]. In a similar study, differences in motor skills persisted in adult mice [17]. Sex-related differences were also observed in mice with tamoxifen-induced microglial depletion; among others, infarct volumes were greater in diphtheria toxin A chain + (DTA+) male mice than in DTA- male mice, whereas no difference was observed in female mice [18]. In the fourth study, male rat neonates did not benefit from TH, in contrast to female rats [19]. Together, these observations underline the urgent need to seek additional neuroregenerative methods in humans, especially for boys who are both at a higher risk of HIE and respond less, if any, to the only available therapy.

## 2. Standard and Experimental Treatment in HIE

The current standard treatment for moderate-to-severe HIE is moderate hypothermia, a temperature decrease of 2–5°C for 72 h, administered within the first 6 h of life [20]. Therapeutic hypothermia (TH) has been shown to decrease the incidence of cerebral palsy in newborns with HIE in randomised clinical trials [21, 22]. Although hypothermia reduces the production of cytokines and metabolic stress, no more than 50% of neonates benefit from this method [11]. TH action is limited only to the superficial parts of the brain, as cooling does not penetrate deep enough to reach its central part. This is reflected in magnetic resonance imaging images, as the basal ganglia appear white in patients treated with TH for up to one month after birth [23]. Therefore, a substantial proportion of infants subjected to hypothermia survive with disabilities. Additionally, possible adverse effects of TH include sinus bradycardia, arrhythmia, hypotension, sepsis, thrombocytopenia, and, in rare cases, reversible subcutaneous fat necrosis [21]. A major contraindication for TH is prematurity owing to the peculiarities of this injury in the preterm brain, as well as a higher incidence of hyperglycaemia, coagulopathy, and mortality in preterm newborns [24, 25]. This strategy was recently verified by Finder et al. [26], who indicated that even mild HIE significantly decreased cognitive ability at a two-year follow-up. In this study, 14 of the 134 children with HIE died. Fifty-five children with mild HIE had lower cognitive composite scores than the control group (mean 97.6 ± 11.9 vs. 103.6 ± 14.6; crude mean difference = 6.0 (95% confidence interval (CI), −9.9 to −2.1), adjusted mean difference = 5.2 (95% CI, −9.1 to −1.3)). No significant difference was observed in the mean cognitive composite scores between untreated children with mild HIE and surviving children with moderate HIE who were treated with TH. A recent study indicated that children with HIE and without cerebral palsy treated with TH are not as school ready as their peers at the age of 5 years due to significantly lower fine motor skills, executive functions, memory, and language than typically developing children [27]. This finding exhibits that even mild HIE has long-term consequences and should not be ignored. Therefore, additional treatment methods for newborns with HIE are urgently needed. In addition to TH, many other drugs have been evaluated for this indication. The leader in this field is the haematopoietic cytokine (erythropoietin (EPO)). The main role of EPO is to prevent the apoptosis of erythroid progenitor cells and to enhance their maturation and proliferation. EPO receptors are expressed by neurons, astrocytes, microglia, and oligodendrocytes and are upregulated in response to brain injury [20]. EPO has neuroprotective and neuroregenerative properties in the brain as it induces anti-inflammatory, antiexcitotoxic, antioxidant, neurogenetic, oligodendrogenesis, angiogenetic, and antiapoptotic effects [28]. Although the elucidation of the role of EPO in signalling after hypoxia won their researchers the Nobel Prize in 2019, its efficacy in clinical studies is limited [20]. Moreover, despite its good general safety profile, EPO may induce high haematocrit, leading to brain injury and disturbing brain development by influencing the proliferation of neuronal stem cells and apoptosis [20]. Reactive oxygen species scavengers appear to be reasonable interventions, consistent with the oxidative pathophysiology of the disease. Some substances in this class, such as melatonin, N-acetylcysteine, docosahexaenoic acid (DHA), edaravone, and allopurinol, have been postulated for this indication. The effectiveness of melatonin (endogenous indoleamine) in HIE appears to be limited [29]. In addition, its neuroprotective action occurs at doses much higher than physiological doses; data regarding its pharmacokinetics, delivery methods, and dosages are scarce [30]. N-acetylcysteine (a precursor of glutathione synthesis and sulfhydryl-containing antioxidant) is likely safe and gives encouraging results, but is not effective in extremely low-birth-weight infants [31]. Edaravone (a low-molecular-weight antioxidant drug that acts an electron donor to peroxyl radicals [32]) was effective in mice administered within 3 h after ischaemic insult when administered alone [33] but did not add any benefits when added to TH in piglet models [34]. Allopurinol is a competitive inhibitor of xanthine oxidase which accumulates in the endothelium of the cerebral capillaries after ischaemic insult, exposing the blood-brain barrier to oxidative stress [35], but this drug has yielded conflicting results [29]. Encouraging the neuroprotective effects of DHA has only been demonstrated in preclinical trials [29]. Therefore, there is an urgent need for new drugs and treatment protocols to improve the prospects of this group of patients. In this review, we aimed to describe the infusion of concentrated autologous cord blood (CB) mononuclear cells (MNC) as a potential therapeutic method for this indication.

## 3. Therapeutic Potential of CB in HIE

CB, a medical waste, is obtained after delivery from an afterbirth. Its noninvasive collection is not related to any risk for a newborn, distinguishing it from alternative haematopoietic stem cell sources such as bone marrow and peripheral blood. In allogeneic settings, it is an attractive alternative in comparison to other hematopoietic stem cell (HSC) sources because of the increased level of human leukocyte antigen disparity that can be tolerated [36, 37]. Moreover, it also offers a lower risk of transmitting latent viral infections and increased proliferative potential compared with adult-derived cells [36], as well as higher immature CD34+ subpopulations [37].

After the separation of red blood cells and plasma, CB mainly comprises regulatory T cells (Tregs), lymphocytes, monocytes, HSC, endothelial progenitor cells, and mesenchymal stem cells (MSC) [38–41]. The therapeutic effect of human CB administered for brain tissue repair and cognitive recovery is dose-dependent in HIE [42, 43]. Transplanted human CB cells have been observed to migrate to the site of injury within 24 h [44]. Mechanisms for the neuroprotective effect of CB stem cells include differentiation into neurons, astrocytes, oligodendrocytes, and microglia [45–49]; antiapoptotic and anti-inflammatory actions [50]; stimulation of angiogenesis; production of trophic factors [51, 52]; and mitochondrial donation [53, 54]. Endothelial progenitor cells decrease neuroinflammation and cell apoptosis [55]. MNC isolated from umbilical CB decrease neuronal apoptosis and inflammation [56] and reduce CD4+ T cell infiltration into the injury site [57]. Monocytes from CB mediate neuroprotective effects by expressing several secreted proteins and by immunoregulation [57–59], and Tregs suppress autoimmunity [60–62]. Human neural progenitors isolated from CB decreased free radical production by 95% and conferred approximately 30% neuroprotection in vitro [63]. In animal models, human CB CD34+ cells administered 48 h after cerebral artery occlusion in mice increased cerebral blood flow and blood vessel diameter in the peri-infarct area [64], probably due to the release of vascular endothelial growth factor and glial-derived neurotrophic factor which stimulates neurogenesis and angiogenesis [65]. MSC can cross the blood-brain barrier and differentiate into neural cells [66, 67]. These cells secrete several factors that reduce oxidative stress, neuroinflammation, and cell death, which translate clinically into improvements in motor and cognitive abilities in a mice model [68]. For example, MSC secretes bFGF [69] which promotes neurological recovery from neonatal HIE by IL-1*β* signalling pathway-mediated axon regeneration [70]. In addition, in the ischaemic brain, MSC can transfer the mitochondria to injured cells [53, 71]. Although the MSC dose in CB is lower than that in medical products based on MSC isolated from umbilical cords which are effective even in children with cerebral palsy [72], the advantage of CB is its immediate availability. However, the disadvantage is an unpredictable cell dose-dependent effect on uncontrollable biological factors. For example, larger amounts of immature haematopoietic progenitors and MSC with high proliferative potential have been detected in CB obtained from preterm newborns than from term newborns [73, 74].

One may wonder whether optimisation of CB therapy should not move toward fractionation of specific cell types. It is not clear to what extent each type of CB cell contributes to the final therapeutic effect. Direct parallel clinical studies comparing the individual fractions for this indication are lacking. Animal studies have shown the clinical equivalence of MNC and HSC [75] as well as MNC and MSC [76] in this field. The MNC fraction contains HSC, endothelial progenitor cells, and mesenchymal stem/stromal cells [77]. These cell populations are defined by the surface marker profile and in vitro growth: HSCs are nonadherent, positive for CD34 and CD133; EPCs are adherent, positive for CD34, CD133, and CD90 and negative for CD13 and CD44; and MSCs are adherent, positive for CD44, CD90, and CD13 and negative for CD34, CD45, and CD133 [77]. Between the 1950s and 1990s, CD34+ cells were considered to be solely haematopoietic; however, as it is shown that CD34+ cells are mobilised from the bone marrow into the peripheral blood circulation in response to ischaemic tissue injury [78], an increasing number of studies have demonstrated their role in regenerative medicine. Administration of these cells was effective in patients with critical limb ischemia [79], myocardial ischemia [80], macular degeneration [81], kidney disease [82], and amyotrophic lateral sclerosis [83]. HIE was effective in animal models, leading to an improvement in cerebral blood flow [84]. The role of MSCs (isolated from both umbilical CB and tissue) in neuroregeneration seems to attract more attention in basic research because they are nonimmunogenic and can be expanded rapidly ex vivo, resulting in the possibility of manufacturing MSC-containing off-the-shelf medicinal products from many tissue sources. CB-derived MSC is an important component of CB, as they reduce the number of apoptotic cells, astrogliosis, and microglial activation, which results in decreased brain damage and improved cognitive and motor function [85]. However, the number of papers describing the importance of MSC in the literature may not reflect the true importance of individual cell fractions in neuroregeneration but may result in effectiveness and feasibility. Endothelial progenitor cells also improved clinical outcomes in animal models, decreased brain damage, and increased the number of mature neurons, cerebral vascularisation, and blood supply to the brain [57, 86, 87]. In mouse models, they showed additional benefits that exceeded those offered by other CB cells [57]. We agree with Ren et al. (who described positive clinical effects after infusion of CBMNC in preterm newborns [88] that more studies on the optimal cell source, including MNC vs. MSC, are needed. To date, some laboratory studies have indicated slight differences between stem cells obtained from different sources. According to Mattar and Bieback [89], who reviewed this discrepancy, MSC obtained from CB have equivalence or advantages over adipose tissue and bone marrow in immunomodulation, proliferation, and senescence; however, the clinical translation of these results remains unknown. Differences between MSC obtained from umbilical CB and tissue are also poorly described, but some studies have reported that a greater number of MSC differentiate into astrocytes and microglia, which translates into better results in animal models of HIE [85].

There may be some doubt as to whether hypoxia adversely affects the CB quality. This issue was assessed recently by Farhat et al. [90], who showed that even severe asphyxia does not negatively affect the viability of CB-derived haematopoietic and progenitor stem cells to grow and form colonies and does not exert negative effects on the quality of CB. Moreover, hypoxia also increases the expression of vascular endothelial growth factor, epidermal growth factor, and fibroblast growth factor, increasing the angiogenic ability of extracellular vesicles secreted by MSC [91].

## 4. CB in Animal Models of HIE

The effectiveness of CB has been repeatedly tested in HIE animal models. In rats, CB MNC administered intraperitoneally 3 h after HI insult resulted in better sensorimotor reflexes, protected neurons in the striatum, and alleviated microglial activation in the cerebral cortex [92]. Administered via the same route 6 h after ischaemic conditions, these cells decreased oxidative stress markers by 36–42%, apoptotic markers by 53–58%, and microglial activation by 51% [93]. This route of administration is justified by the high vascularisation of the peritoneum which allows cells to access the lymphatic and blood circulatory systems simultaneously [94]. Meier et al. [95] demonstrated the presence of transplanted human CB cells in damaged brain lesions 3 days and 2 weeks after intraperitoneal injection, although these cells did not overlap with neural markers, indicating that they did not differentiate into neural cells. After this route of administration, fewer cells were found in the spleen and lungs than after systemic injection, and the treatment effect might be caused by trophic factors secreted by the cells rather than tropism and homing [94].

When injected into the lateral ventricle, MNC reduced astrogliosis, prevented the neuronal loss in the striatum, increased the proportion of mature oligodendrocytes, improved myelination in the cortex, and improved functional brain outcomes after a 28-day recovery period [76]. In rats, human umbilical CBMSC attenuated neuronal loss and promoted neurogenesis in the hippocampus of neonatal rats with intraventricular haemorrhage [96]. The authors who indicated that a single CB infusion was not effective in a rat model of HIE later showed that repeated infusions of CB improved motor strength and limb asymmetry, behaviour (short-term memory and exploratory behaviours), brain weight, and neuron numbers in the motor cortex and somatosensory cortex and reduced apoptotic and inflammatory markers [97].

Interestingly, Yu et al. [75] compared CB MNC and CB CD34+ (haematopoietic) cells in a rat model of HIE. Administration of CD34+ cells significantly improved motor function, reduced cerebral atrophy, and decreased the expression of the glial fibrillary acidic protein, a marker of gliosis, and five apoptosis-related genes. It also increased the expression of doublecortin (a neuronal migration protein) and lectin (a marker of microvascular density). The authors indicated that the transplantation of CD34+ cells and MNC obtained from the same amount of human umbilical CB had similar effects. Neuroprotection after systemic delivery of stem cells is independent of their entry into the central nervous system, probably due to soluble factors [98, 99]. This observation suggests that haematopoietic stem cells are mainly responsible for the neuroregenerative properties of CB. Previously, the therapeutic effect of this cell population was demonstrated in a mouse model of stroke [100, 101].

In a mouse model, infiltration of CD4+ T cells into the injured cerebral hemisphere was significantly reduced by all human CB cell types. Tregs additionally reduced motor deficits, CD4+ T cell infiltration into the brain, and microglial activation [57].

In sheep, human CB protects the white matter, decreases inflammation, and recovers total and mature oligodendrocytes [102]. In another lamb model, the administration of CB MNC 12 h after perinatal asphyxia reduced neuroinflammation, astrogliosis, and neuronal apoptosis within the basal ganglia, thalamus, hippocampus, cortex, and subcortical white matter; the restoration of normal brain metabolic activity within the basal ganglia, thalamus, and hippocampus was confirmed using magnetic resonance spectroscopy [103]. In a preterm sheep model of white matter injury, infusion of CB-derived MSC administered after hypoxia-ischaemia in the preterm brain reduced white matter injury by inhibiting microglial activation and the release of TNF*α*, promoting macrophage migration, and accelerating self-repair [89, 104, 105].

These observations translate to clinical changes. Long-term studies have shown that even a single dose of CB improves gait [106], locomotion [84], cognitive function [107–119], anxiety [110], and limb use [86, 106, 109–110].

## 5. Clinical Studies

The efficacy and safety of CB administration were reported in a few early clinical studies. Cotten et al. [111], who administered noncryopreserved autologous volume- and red blood cell-reduced umbilical CB cells to neonates with HIE, indicated that this procedure is possible and safe. Moreover, these authors observed numerically better survival (74% vs. 41%, although the difference was not significant, probably due to the low number of participants) and development (72% vs. 41% surviving with all three Bayley domain scores ≥85) in cell recipients than in the control group treated with TH [84]. Yang et al. [112] and Kotowski et al. [113] reported similar results in preterm and very premature neonates, respectively, using the same cell fraction. Kotowski et al. noticed a significant increase in 22 plasma proteins and a decreased percentage of intraventricular haemorrhages in the group treated with CB (40% vs. 86.7%). In 2020, Tsuji et al. [114] described the results of CB use in six newborns with severe asphyxia who received CB within 24–72 h after birth. At 30 days of age, all of them survived without circulatory or respiratory support; at 18 months of age, two had cerebral palsy; and in four infants, neurofunctional development was normal according to the Kyoto Scale of Psychological Development.

The longest follow-up after a single CB injection in newborns at risk for encephalopathy was published in 2020 by Zhuxiao et al. [115]. We observed 15 very and moderately preterm newborns (28–34 weeks of gestation) for 10 years after delivery to assess the long-term safety, growth, respiratory, and neurodevelopmental outcomes of these enrolled patients. Although none of these patients had an ischaemic insult, preterm delivery is a risk factor for negative neurological outcomes [116]. None of the patients had cerebral palsy or auditory or visual impairment. The average intelligence quotient was 95.27, and no patients were diagnosed with mental disabilities. None of the children had any cancer or tumours.

Currently, there are two ongoing interventional clinical trials registered under US regulations assessing CB use in children with HIE: NCT02434965 (phase 2, single-arm, open-label, 20 participants with severe HIE) and NCT02551003 (phase 2, two-arm, single-blinded, 60 participants). In addition, in the United States, CB is being used in the expanded access program as an Investigational New Drug. From November 2017 to June 2019, 276 children, including those with cerebral palsy, received 302 CB infusions under the expanded access protocol [117]. Early but encouraging results of these interventions were presented at the Cord Blood Connect conference in 2020.  Table 1 shows the cord blood elements that may contribute to neuroregeneration.

## 6. Candidates for CB Administration

Currently, the diagnosis of HIE is based on existing neuroimaging abnormalities, even though mild HIE is associated with poorer neurological prognosis in long-term observation. The severity of HIE depends on the course of the asphyxiating insult, gestational age, prior metabolic and cardiovascular status, and individual sensitivity to oxidative stress [118]. The combination of clinical signs remains the main basis of HIE severity classification [119], although clinical signs and cerebral electrical activity may be altered by drugs and TH [120]. Therefore, predictors are sought to qualify newborns for early therapeutic intervention. Well-known prenatal risk factors for HIE include maternal pyrexia, prolonged rupture of membranes, persistent occipito-posterior position, placental abruption, prolapse of the umbilical cord, uterine rupture, and shoulder dystocia [121, 122]. After birth, increased HIE risk was associated with umbilical cord arterial pH < 6.8, base excess < −20 mEq/L, Apgar score ≤ 3 at 10 min, absence of foetal heart rate variability before birth, seizures on the first day of life, and multiorgan injury [118]. Recently, an increasing number of publications have described the prognostic role of markers of brain injury associated with HIE [123–126]. However, to date, although some studies have described the predictive value of the interleukin profile or metabolic parameters [127–129], no single marker has demonstrated sufficient reliability and reproducibility to be used as a biomarker or therapeutic target in a clinical setting even at the epigenetic level [130]. So far, the greatest area under the curve (0.96) resulted in a model which combines clinical signs with lactate and alanine levels [131]. Alternatively, CB may be collected from at-risk children; therefore, it can be applied if delivery is complicated by HIE, or even prophylactic administration in children with risk factors may be considered due to the low risk associated with the infusion of autologous CB.

In addition to the eligibility criteria, the second issue is the timing. Animal studies have revealed the significance of stem cell administration timing, which is crucial for the effectiveness of neuroregenerative therapy. In a study assessing the preventive administration of caffeine, which shows antioxidative properties, therapy was effective only if administered directly after the insult [132]. TH is effective within the first 6 h [133]. According to some authors, the therapeutic window for cellular therapy can be as long as 12 h [56] or even 3 days [134]. TH may also broaden the therapeutic window for stem cell therapy [135]. All of these data come from animal models; thus, the effective use of this promising therapeutic method in children certainly requires further optimisation. In particular, further evaluation is needed to balance the risk resulting from a delay in therapy due to the need to test CB for infectious agents and the risk of disability caused by a lack of early intervention.

The limitations of our review are its narrative character and search strategy limited to the English language and PubMed database, provided by a single person. Despite these limitations, the use of CB seems to be a rational and promising experimental therapy that requires further investigation, especially in the field of future optimisation and development of therapeutic schemes involving CB.

## Figures and Tables

**Table 1:** Cord blood elements that may contribute to neuroregeneration.

Fraction	Potential role in brain regeneration	Reference
Hematopoietic stem cells	Release of vascular endothelial growth factor and glial-derived neurotrophic factor which stimulate neurogenesis and angiogenesis	[65]
Mesenchymal stem cells	Reduce oxidative stress, neuroinflammation, and cell death; transfer mitochondria to injured cells	[68–71]
Regulatory T cells	Suppress autoimmunity	[60–62]
Monocytes	Mediate the neuroprotective effects via expressing several secreted proteins and immunoregulation	[57–59]
Progenitors	Decrease neuroinflammation, cell apoptosis, and free radical production	[55, 63]
